# Pitfalls in Bacteriological Diagnosis

**Published:** 1897-04-24

**Authors:** 


### PITFALLS IN BACTERIOLOGICAL DIAGNOSIS.

The difficulties which are likely to be caused by a too great readiness to place trust in bacteriological tests rather than in clinical evidences of disease are well illustrated in the case of gonorrhœa. It is maintained that a secretion may be present in the vagina which bears an exact resemblance macroscopically to gonorrhœal pus, but yet contains no gonococci. On the other hand it is admitted that a secretion may be virulent, although it may contain but a few scattered cocci, which are only to be found after a long search. Everyone knows clinically that acute attacks may be set up by infection from cases which have seemed to be quite cured, and if in these apparently cured cases gonococci were plainly demonstrable so long as they remained infective and no longer, bacteriological examination would indeed be of much practical service. This, however, does not seem to be the case. These organisms are too difficult of demonstration in the very cases where their demonstration would be most useful to permit of their apparent absence being accepted as proof of cure, while their presence is so common among careless livers, that it is hardly justifiable to attach very much importance to them in medico-legal inquiries, as, for example, in accusations of rape.

